# Colorectal Cancer in Young Patients: Experience From Avicenne Military Hospital, Marrakech

**DOI:** 10.7759/cureus.95998

**Published:** 2025-11-03

**Authors:** Moussa Abdoul Aziz Sawadogo, Soukayna Boujmadi, Hamza Laabbar, Mohamed Kaakoua, Ismail Essadi

**Affiliations:** 1 Medical Oncology, Ibn Sina Military Hospital, Marrakech, MAR; 2 Medical Oncology, Avicenne Military Hospital, Marrakech, MAR

**Keywords:** cancer, colorectal cancer, colorectal cancer in morocco, colorectal cancer in young adults, young adults

## Abstract

Background: Colorectal cancer (CRC) in young adults is an increasingly significant global health concern. Several studies have documented a rise in its incidence and tumor aggressiveness, yet no specific management recommendations exist for this age group. This study aims to describe the clinical, therapeutic, and prognostic characteristics of young patients treated for CRC at our center.

Methods: This retrospective study was conducted at the Medical Oncology Department of Avicenne Military Hospital in Marrakech, covering the period from January 2019 to December 2023. Twenty-eight patients under 50 years of age diagnosed with CRC were included and analyzed.

Results: Among the 28 patients, the mean age at diagnosis was 31.9 years, with a slight male predominance (n = 15; sex ratio 1.15). Four patients were active smokers (14%), one was a chronic alcohol user, and two had a family history of CRC (7%). The most frequent presenting symptoms were abdominal pain (11 cases, 39%), constipation (8 cases, 28%), bowel obstruction (6 cases, 21%), and gastrointestinal bleeding (3 cases, 10%). Tumor location was predominantly in the right colon (n = 15, 53.5%), followed by the left colon (n = 11, 39.2%) and the rectum (n = 2, 7.1%). The most common histological type was Lieberkühnian adenocarcinoma, found in 71.5% of cases (n = 20). Among metastatic patients, the median progression-free survival was 25 months.

Conclusion: CRC in young adults presents an aggressive clinical profile. Improved outcomes require earlier diagnosis, particularly in patients with predisposing factors, and the development of specialized expert centers for optimal management.

## Introduction

Colorectal cancer (CRC) is the most common digestive malignancy. It ranks as the third most frequent cancer and the second leading cause of cancer-related death worldwide [1]. In recent years, its incidence among young adults has been steadily increasing. The average age at diagnosis is 50-52 years in Morocco and rarely below 50 years in Western countries (6% of cases). In Morocco, 25% of patients are under 40 years in most hospital series [2]. In the absence of specific recommendations for this population, management remains a major challenge. This study aims to describe the clinical, therapeutic, and prognostic characteristics of young patients treated for CRC in our center.

## Materials and methods

This retrospective study was conducted at the Medical Oncology Department of Avicenne Military Hospital in Marrakech. It included all patients aged between 18 and 50 years who were diagnosed with CRC, regardless of disease stage at diagnosis, and followed between January 2019 and December 2023. Data were collected from medical records, including clinical observations, laboratory results, CT scans, scintigraphy, and histopathological examinations with immunohistochemical findings. Data collection was based on primary records available in the department. For each patient, the following parameters were recorded: name, sex, age, origin, medical, family, and surgical history, toxic habits, clinical presentation, tumor location, disease stage, histological type, presence of lymphadenopathy, vascular emboli, perineural invasion, and resection margins. Incomplete or missing records were excluded from the study. CRC was defined as any malignancy arising from the walls of the right colon, left colon, or rectum. 

## Results

**Figure 1:**
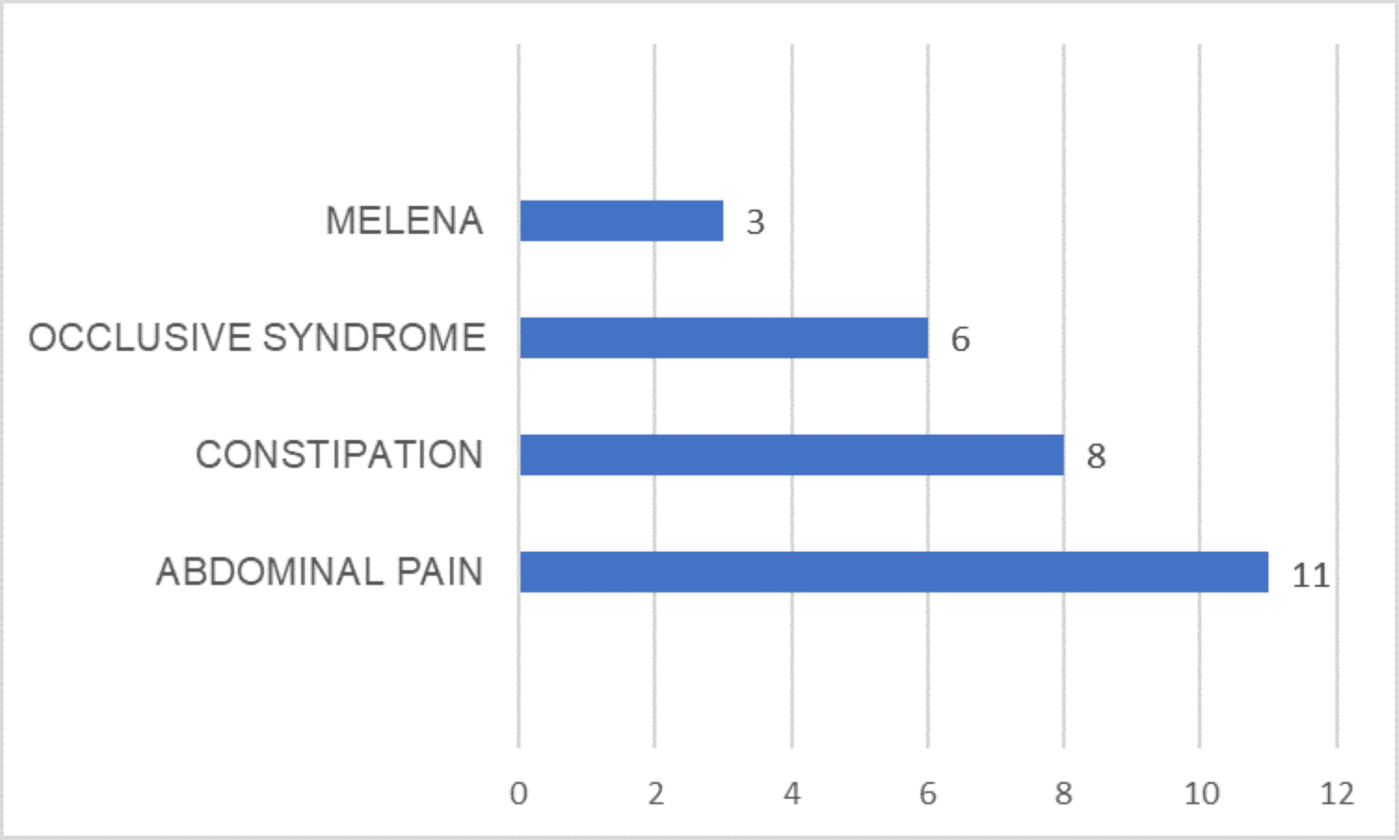
Distribution of disease symptoms (n = 28).

Among 108 patients followed for CRC during this period, 28 patients (25%) were under 50 years of age, with a mean age at diagnosis of 32 years (range: 22-47 years). There was a slight male predominance (n = 15), with a sex ratio of 1.15. Four patients were active smokers (14%), one was a chronic alcoholic, and two had a family history of colorectal cancer (7%). The most common presenting symptoms were abdominal pain (11 cases, 39%), constipation (8 cases, 28%), occlusive syndrome (6 cases, 21%), and gastrointestinal bleeding (3 cases, 10%) (Figure 1).

**Figure 2:**
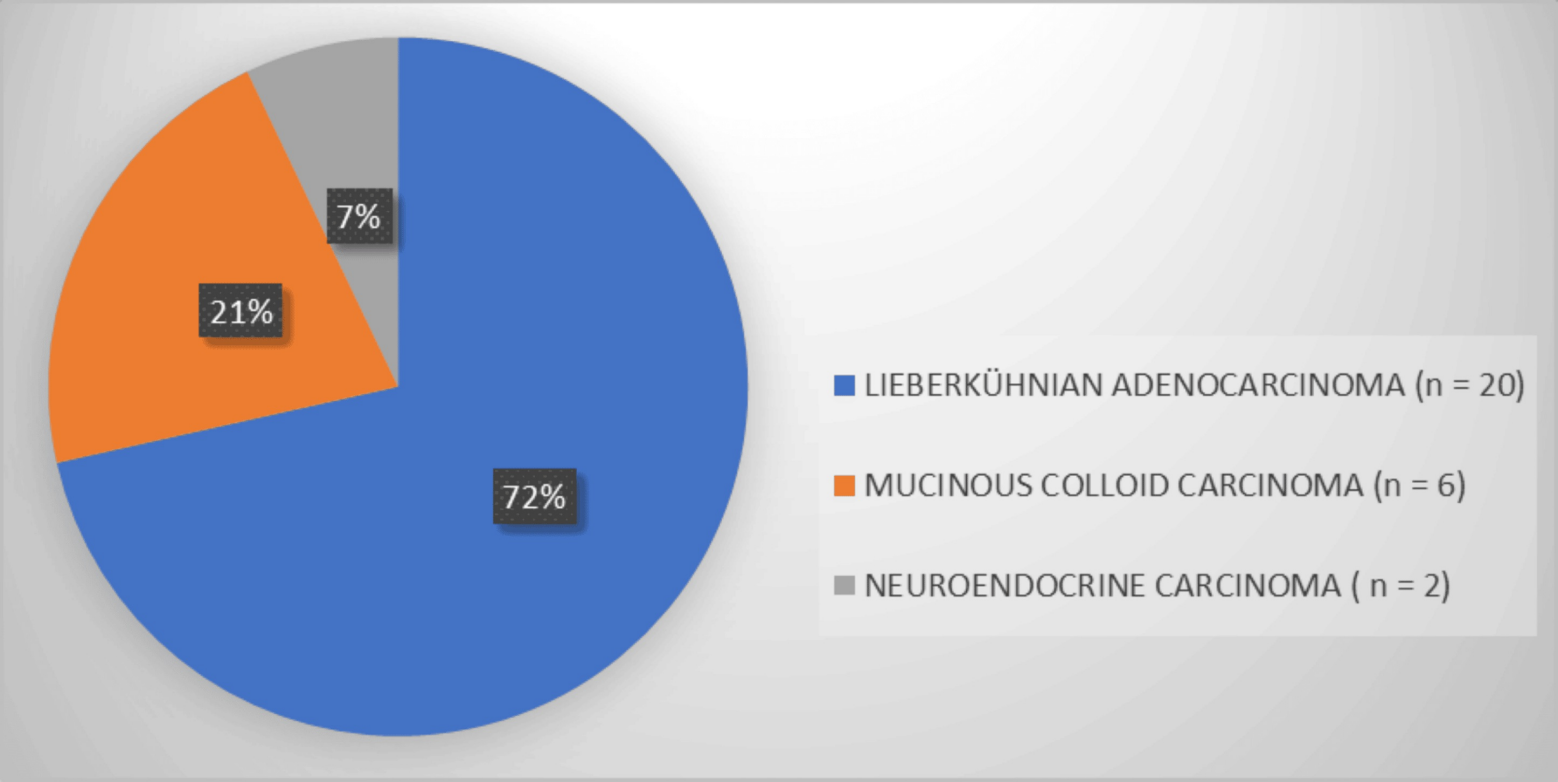
Distribution of patients according to histological type (n = 28).

In 54% of cases, the cancer was located in the right colon (n = 15), 39% in the left colon (n = 11), and 7% in the rectum (n = 2). The most common histological type was Lieberkühnian adenocarcinoma, observed in 72% of cases, followed by mucinous (colloid) adenocarcinoma in 21% and neuroendocrine carcinoma in 7% (Figure 2).

**Figure 3:**
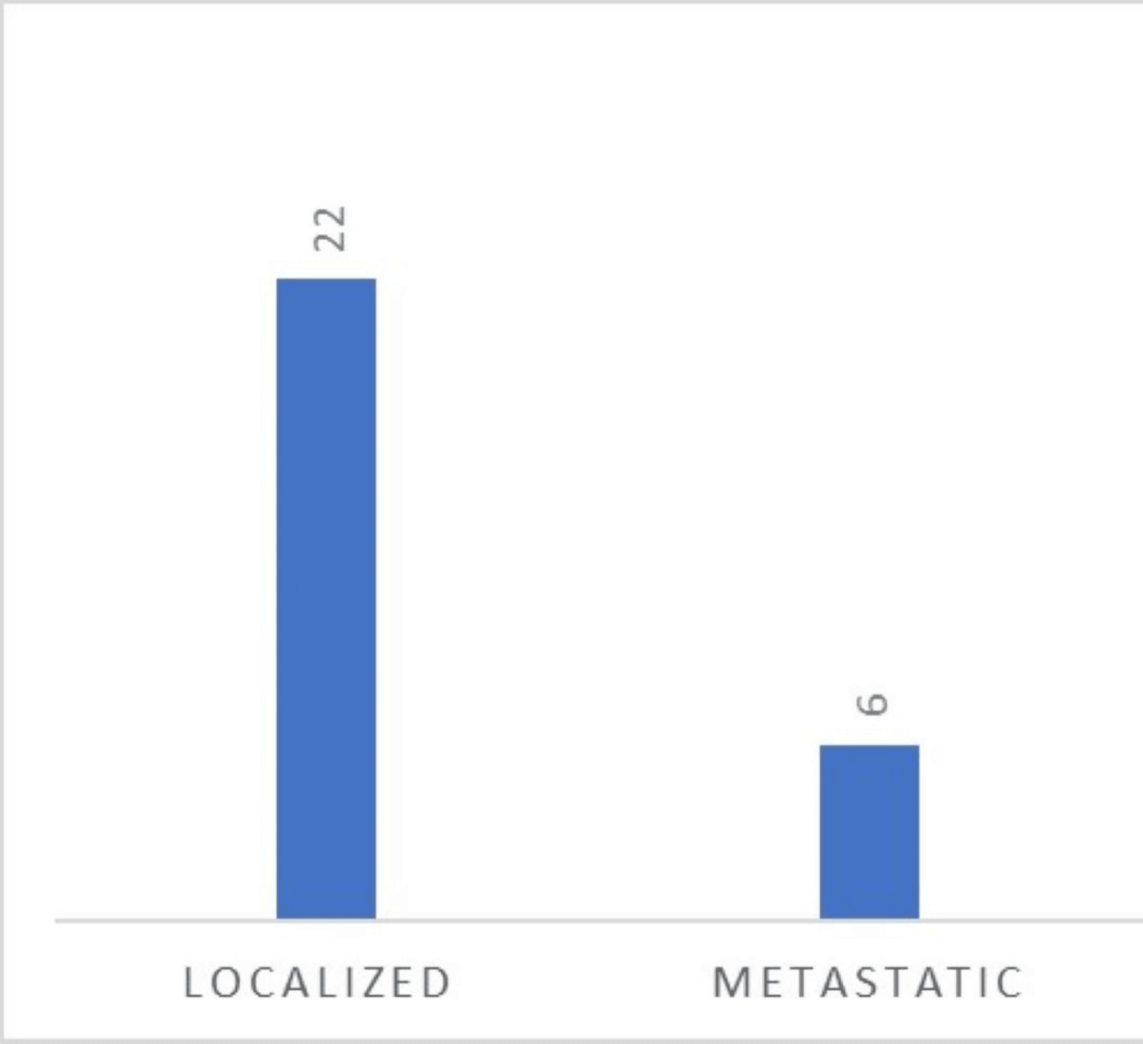
Distribution of patients according to the stage of the disease (n = 28).

At diagnosis, CA 19-9 and CEA levels were elevated in 25% and 36% of cases, respectively. The disease was classified as Stage I in nine cases (32%), Stage II in five cases (18%), Stage III in eight cases (29%), and Stage IV in six cases (21%). Among the metastatic patients, four presented with liver metastases and three with peritoneal metastases. Adjuvant chemotherapy was recommended for one Stage II patient due to poor prognostic factors, including T4 stage, elevated preoperative CEA, and the presence of vascular emboli. All Stage III patients received adjuvant chemotherapy: five received three months of XELOX, while the remaining three received six months of FOLFOX. Of the six metastatic patients, two had wild-type RAS and BRAF status. Four were treated with a chemotherapy-bevacizumab combination, one with a chemotherapy-cetuximab combination, and one underwent hyperthermic intraperitoneal chemotherapy (HIPEC). After a median follow-up of 36 months, the relapse rate among patients with localized disease (n = 22) was 9%, with two patients developing liver recurrence (Figure 3).

For metastatic patients, the median progression-free survival was 25 months, with only one reported death.

## Discussion

CRC is the most common digestive cancer in terms of incidence [3]. The median age of onset is 74 years, with more than 94% of cases occurring after the age of 50 [4]. The proportion of CRC before 50 years is estimated at 6% [4]. However, recent studies have reported a worrying increase in this proportion, now reaching up to 20% [5]. In our series, the proportion was even higher, at 25%.

The mean age in our study was 32 years, which aligns with the literature, where it generally varies between 31 and 40 years [4-6]. A slight male predominance was observed, consistent with findings from several other series [7,8].

Regarding the presenting symptoms, previous studies have reported rectal bleeding in around 40% of cases, abdominal pain in 25%-35%, and bowel transit disorders in 24% [8-11].

Several studies have shown that CRC in young patients tends to be located predominantly in the left colon, which may contribute to the aggressive nature of these cancers [6,8]. However, our findings differed, showing a predominance of right-sided colon cancer, consistent with the results reported by Pocard et al. [7].

The most frequent histological type in our study was adenocarcinoma (71.5%), similar to Ouedraogo et al., who reported 88.7% [6]. Other studies have noted a higher frequency of rectal and sigmoid involvement in young patients [12,13]. In our series, advanced stages (Stage III or IV) represented 50% of cases at diagnosis. This high frequency of aggressive forms in young subjects is consistent with previous reports describing poorer prognoses in this age group [6,10].

In younger patients, delayed diagnosis may be due to the fact that CRC is rarely suspected initially because of its low prevalence in this population. Unfortunately, diagnosis is often only made once symptoms become significant, usually corresponding to an advanced stage. Therefore, it may be necessary to include younger individuals in systematic screening programs similar to those for older populations [6].

For localized stages, the recurrence rate in our study was 9%, which is comparable to the 11.1% reported by Soualili et al. [14]. Unfortunately, we were unable to perform further analyses on genetic predisposition.

For metastatic stages, the median progression-free survival was 25 months, longer than that observed in the older population reported by Koopman et al. [15].

Our study faced some limitations. Some patients were lost to follow-up or had died by the time of data collection, which limited the completeness of our dataset. In addition, genetic testing and oncogenetic consultations were not performed, which restricted our ability to explore hereditary or molecular risk factors.

## Conclusions

CRC in young patients is characterized by an aggressive profile, as shown in this preliminary study, with symptoms comparable to those observed in older individuals. This aggressiveness warrants greater clinical attention to improve prognosis and optimize treatment strategies, particularly through the identification of familial forms in the presence of predisposing factors. More extensive and representative studies are needed to better understand the specific features of CRC in this population. Furthermore, the development of specialized cancer units capable of providing comprehensive care for young patients is essential. Preventive measures and early management should also be prioritized to reduce disease burden in this age group.
